# Hydroxychloroquine and Coronavirus Disease 2019: A Systematic Review of a Scientific Failure

**DOI:** 10.5041/RMMJ.10416

**Published:** 2020-07-31

**Authors:** Stav Rakedzon, Yara Khoury, Gilad Rozenberg, Ami Neuberger

**Affiliations:** 1 Department of Internal Medicine, Rambam Health Care Campus, Haifa, Israel; 2 Division of Infectious Diseases, Rambam Health Care Campus, Haifa, Israel; 3 The Ruth & Bruce Rappaport Faculty of Medicine, Technion–Israel Institute of Technology, Haifa, Israel

**Keywords:** Chloroquine, coronavirus, COVID-19, hydroxychloroquine, pandemic, review

## Abstract

**Introduction:**

Hydroxychloroquine (HCQ) emerged early in the course of the coronavirus disease 2019 (COVID-19) pandemic as a possible drug with potential therapeutic and prophylactic benefits. It was quickly adopted in China, Europe, and the USA. We systematically reviewed the existing clinical evidence of HCQ use for the prevention and treatment of COVID-19.

**Methods:**

We screened for clinical studies describing HCQ administration to treat or prevent COVID-19 in PubMed. We included randomized controlled trials (RCTs), non-randomized comparative cohorts, and case series studies that had all undergone peer review.

**Results:**

A total of 623 studies were screened; 17 studies evaluating HCQ treatment were included. A total of 13 were observational studies, and 4 were RCTs. In terms of effect on mortality rates, observational studies provided conflicting results. As a whole, RCTs, including one large British RCT that has not yet been published, showed no significant effect of HCQ on mortality rates, clinical cure, and virologic response. The use of HCQ as a post-exposure prophylactic agent was found to be ineffective in one RCT.

**Conclusion:**

There is no evidence supporting HCQ for prophylaxis or treatment of COVID-19. Many observational trials were methodologically flawed. Scientific efforts have been disappointingly fragmented, and well-conducted trials have only recently been completed, more than 7 months and 600,000 deaths into the pandemic.

## INTRODUCTION

The coronavirus disease 2019 (COVID-19) pandemic is a global healthcare crisis that has already cost more than 600,000 lives worldwide, and is associated with enormous economic, geopolitical, and social burdens.^1^

As no drug was known to be effective at the onset of the pandemic, efforts were aimed at repurposing existing agents to treat severe acute respiratory syndrome coronavirus-2 (SARS-CoV-2). Very early on in the course of the pandemic, chloroquine and its less toxic derivative hydroxychloroquine (HCQ) emerged as possible candidates and were quickly adopted in China, where COVID-19 had initially emerged.

Chloroquine and HCQ have traditionally been used to treat chronic inflammatory diseases, malaria, and Q fever. *In vitro* SARS-CoV-2 inhibition was thought to be caused by interference with viral entry to host cells and the reduction of viral replication.^2^–^5^ Additionally, established immunomodulatory effects of HCQ were considered to be potentially beneficial in treating patients suffering “cytokine storm” due to COVID-19. As the pandemic progressed, the higher potency of HCQ against SARS-CoV-2 (as compared to chloroquine),^6^,^7^ together with its improved safety profile, made HCQ part of national guidelines in many countries. A heated medical and political debate regarding the theoretical benefits and possible adverse events of HCQ use for COVID-19 has been going on for months. Studies have been retracted from major medical journals, and authors were blamed for methodological inconsistencies and lack of transparency. Even now, 7 months into the global pandemic, conclusive studies have not yet been published.

This paper therefore provides a systematic review of the existing clinical evidence regarding the use of HCQ for the prevention and treatment of COVID-19, and we discuss some of the factors that led to this failure of the scientific community.

## METHODS

### Type of Studies

For the purpose of review, we looked for clinical studies that administered HCQ to treat or prevent COVID-19. Inclusion criteria were: patients of all ages who had been diagnosed with COVID-19 and were treated with hydroxychloroquine, and were participants in randomized controlled trials (RCTs), non-randomized comparative cohort studies, and/or case series studies. Any study not meeting the above criteria, or that included patients who did not have COVID-19, or were taking hydroxychloroquine for other indications, and/or infections other than COVID-19, were excluded.

### Types of Outcome Measures

Clinically relevant outcomes such as mortality rates, rates of admission, length of hospital stay, need for mechanical ventilation, and disease severity were noted. In addition, we included virologic parameters such as viral clearance times, viral shedding duration, and viral load.

### Search Methods for Identification of Studies

We conducted a systematic electronic literature search within PubMed. We used one of the following search terms: “COVID-19” and/or “coronavirus,” combined with the drug name “hydroxychloroquine.” We also used the references of retrieved papers, including reviews and systematic reviews, to identify further studies. Two reviewers (authors S.R. and G.R.) independently screened all studies published before 20 June 2020. We excluded all initially identified and retrieved articles that did not fulfill the inclusion criteria. In case of disagreement, a third reviewer acted as arbitrator (author A.N.). Reporting was performed in accordance with the Preferred Reporting Items for Systematic Review and Meta-Analyses (PRISMA).

## RESULTS

### Studies Evaluating HCQ for the Treatment of COVID-19

Our systematic search yielded 623 studies from PubMed. Overall, we included 13 observational studies describing outcomes of 8,967 patients treated with HCQ (Table 1)^8^–^20^ and 4 RCTs (one of which was a preliminary report of an unpublished study^22^) that described the outcomes of 2,299 patients treated with HCQ (Table 2).^23^–^25^,^37^ We describe separately several studies (apart from the 17 studies that were formally included in the analysis) with severe methodological issues (Table 3).^26^–^29^

**Table 1:** Summary of Observational Studies Evaluating HCQ for COVID-19 Treatment.

Study Design	Pts	Study Groups	Results: Primary/Secondary* Outcomes	Methodological Issues
Comparative observational study^18^	3737	1. HCQ+AZ 2. Others sub divided to: HCQ-AZ <3 days HCQ AZ SOC	HCQ+AZ treatment compared to control: ↓ Risk of death or transfer to ICU (HR 0.18; 95% CI 0.11–0.27)* ↓ Hospitalization ≥10 days (OR 0.38; 95% CI 0.27–0.54) Shorter viral shedding duration in HCQ+AZ compared with all other subdivided groups, compared with SOC (HR 1.29; 95% CI 1.17–1.42)	Unequal group sizes (Tx Pts *n*=3,119 vs “Other” Pts *n*=618; with many Txs and no pre-specified protocol
Comparative observational study^15^	2541	1. HCQ 2. HCQ+AZ 3. AZ 4. SOC	Overall in-hospital mortality lower in all treatment groups: HCQ+AZ: 20.1% (95% CI 17.3%–23.0%) HCQ: 13.5% (95% CI 11.6%–15.5%) AZ: 22.4% (95% CI 16.0%–30.1%) SOC: 26.4% (95% CI 22.2%–31.0%)	Tx group Pts mean age 5 y older than in controls ↑ steroid Tx rates in HCQ groups Missing data due to reliance only on electronic health records
Comparative observational study^10^	1438	1. HCQ 2. HCQ+AZ 3. AZ 4. SOC	No Sig.Dif. in mortality rates for HCQ+AZ (HR 1.35; 95% CI 0.76–2.40), HCQ alone (HR 1.08; 95% CI 0.63–1.85), or AZ alone (HR 0.56; 95% CI 0.26–1.21) compared with control*	No follow-up of discharged Pts Missing group characteristics data Major difference between size of SOC vs Tx groups
Comparative observational study^12^	1376	1. HCQ 2. SOC	No significant association between HCQ and intubation or death vs SOC (HR 1.04; 95% CI 0.82–1.32)	HCQ Pts more severely ill than SOC at baseline
Comparative observational study of critically ill ventilated patients with ARDS^14^	568	1. HCQ 2. SOC	**Primary Outcome** Significant mortality rate differences between HCQ (18.8%) and SOC (45.8%) groups; *P*<0.001 **Secondary Outcomes** Longer HCQ hospitalization time before death compared to SOC (*P*<0.05) IL-6 levels significantly lower during Tx period for HCQ group; SOC group unchanged	Such dramatic ↓ in mortality rate not described in any other study Different rates of antibiotics use and interferon imply inherent selection bias for HCQ vs SOC (HCQ 0% interferon vs 10.8% in SOC (*P*=0.01)
Comparative observational study^16^	72	1. HCQ-asymptomatic 2. SOC-asymptomatic	No Sig.Dif. in recovery rates (HCQ 97.5% vs SOC 96.85%) Earlier Tx group recovery (5.4 days) vs SOC (7.6 days)	HCQ efficiency only assessed in asymptomatic Pts Inclusion to SOC due to HCQ contraindications that probably imply underlying medical conditions
Comparative observational study of only electronic health records^13^	368	1. HCQ 2. HCQ+AZ 3. SOC	**Primary Outcomes** ↑ mortality rates for HCQ (AHR 2.61; 95% CI 1.10–6.17; *P*=0.03) vs SOC, but not in HCQ+AZ (AHR 1.14; 95% CI 0.56–2.32; *P*=0.72) No Sig.Dif. in ventilation rates among HCQ, HCQ+AZ, and SOC (13.3%, 6.9%, 14.1%, respectively) **Secondary Outcomes** No difference in risk of death after ventilation in HCQ (AHR 4.08; 95% CI 0.77–21.70; *P*=0.10), HCQ+AZ (AHR 1.20; 95% CI 0.25–5.77; *P*=0.82), compared with the no HCQ group	Missing data due to reliance on electronic health record codes ~95% males in all groups
Comparative observational study of pneumonia patients requiring O_2_ without ICU admission^11^	181	1. HCQ 2. SOC	**Primary Outcome** No Sig.Dif. in survival rates at day 21 for Pts not transferred to the ICU; HCQ 76% vs SOC 75% (WHR 0.9; 95% CI 0.4–2.1) **Secondary Outcomes** No Sig.Dif. in overall survival at day 21: HCQ (89%) vs SOC (91%) (WHR 1.2; 95% CI 0.4–3.3) No SC for O_2_ weaning at day 21: HCQ 82% vs SOC 76% (WRR 1.1; 95% CI 0.9–1.3)	HCQ Pts with fewer comorbidities No Tx allocation protocols
Comparative observational study^17^	84	1. HCQ 2. SOC	No Sig.Dif. for: Reducing unfavorable outcome risk (defined as death, ICU admission, or decision to withdraw or withhold life-sustaining treatments) (HR 0.90; 95% CI 0.38–2.1; *P*=0.81) Overall survival (HR 0.89; 95% CI 0.23–3.47; *P*=1)	Small-scale study
Comparative observational study^19^	65	1. HCQ 2. Lopinavir-ritonavir	**Primary Outcome** Significantly shorter time to virological cure in lopinavir-ritonavir group (median 21 days vs 28 days; *P*=0.029) **Secondary Outcome** No Sig.Dif. in time to clinical improvement between groups (median 18 days vs 21 days; *P*=0.216)	More pneumonia Pts in lopinavir-ritonavir group
Comparative observational study^20^	60	1. Azithromycin, prednisolone, naproxen, and lopinavir/ritonavir 2. Meropenem, levofloxacin, vancomycin, HCQ, and oseltamivir	**Primary Outcome** SpO_2_ saturation, body temperature, and CRP values more favorable in Group 1 Pts (*P*=0.013, *P*=0.012, *P*<0.001, respectively). **Secondary Outcome** Significantly ↓ length of hospitalization for Group 1 vs Group 2 (*P*=0.001)	Due to multiple drug regimens, unclear relative contribution of each drug to the results Adding broad-spectrum antibiotic to COVID-19 is probably unnecessary^21^
Uncontrolled observational study^9^	1061	1. HCQ+AZ	Poor clinical outcome for 46 Pts (4.3%), 8 died (0.75%), 6 transferred to ICU (0.56%); 30 hospital stays >10 days (2.8%) Prolonged viral carriage in 47 Pts (4.4%)	Uncontrolled study Relatively young Pts: mean age 43.6 y
Uncontrolled observational study^8^	80	1. HCQ+AZ	Clinical improvement: 97.5% Pts Mean length of hospital stay: 5 days Negative virus cultures in 97.5% Pts at day 5	Uncontrolled study Inadequate length of post-treatment follow-up Relatively young Pts: median age 52.5 y

* Only primary outcomes are shown, unless the study also provided secondary outcomes.

↓, decreased/lower; ↑, increased/higher; AHR, adjusted hazard ratio; ARDS, acute respiratory distress syn-drome; AZ, azithromycin; CI, confidence interval; CRP, C-reactive protein; HCQ, hydroxychloroquine; HR, haz-ard ratio; ICU, intensive care unit; O_2_, oxygen; Pts, patients; Sig.Dif., significant difference(s); SOC, standard of care; SpO_2_, oxygen saturation; Tx, treatment group; WHR, weighted hazard ratio; WRR, weighted risk ratio.

**Table 2:** Summary of Randomized Control Trials (RCT) for Evaluating HCQ for COVID-19 Treatment.

Study Design	Pts	Study Groups	Results: Primary/Secondary Outcomes	Methodological Issues
RCT^24^	150	1. HCQ 2. SOC	No Sig.Dif. in virological cure rates at day 28	Open-label study
RCT^23^	30	1. HCQ 2. SOC	**Primary Outcome** No Sig.Dif. in virological cure rates (HCQ 86.7% vs SOC 93.3%; *P*>0.05) **Secondary Outcome** No Sig.Dif. for time to body temperature normalization and improvement in follow-up examinations	Published only in Chinese Small-scale study
Preliminary data from RCT^22^	4674	1. HCQ 2. SOC	**Primary Outcome** No Sig.Dif. in survival rates at 28 days (25.7% vs 23.5% in treatment versus SOC, HR 1.11 [95% CI 0.98–1.26]; *P*=0.10) **Secondary Outcome** No impact of HCQ Tx on length of hospitalization	Preliminary results; not yet peer-reviewed
RCT^25^	667	1. HCQ 2. HCQ+AZ 3. SOC	**Primary Outcome** No clinical status benefit at 15 days **Secondary Outcome** No mortality benefit For HCQ Pts and HCQ+AZ: Increased incidence of QTc prolongation and elevated liver enzymes in both treatment groups when compared to SOC	

AZ, azithromycin; CI, confidence interval; HCQ, hydroxychloroquine; HR, hazard ratio; Sig.Dif., significant difference(s); SOC, standard of care.

**Table 3:** Studies with Severe Methodological Issues Evaluating HCQ for COVID-19 Treatment.

Study Design	Pts	Study Groups	Results: Primary/Secondary* Outcomes	Methodological Issues
Comparative multinational registry analysis^29^	96,032	1. CQ 2. CQ+macrolide 3. HCQ 4. HCQ+macrolide 5. SOC	**Primary Outcome** Significant ↑ of in-hospital mortality rates for CQ (16.4%); CQ+macrolide (22.2%); HCQ (18.0%); HCQ+macrolide (23.8%); vs SOC (9.3%) **Secondary Outcome** All Tx groups independently associated with ↑ risk of *de novo* ventricular arrhythmia during hospitalization	Publication retracted as per request of three co-authors The corporation that initially provided the Pt dataset refused to transfer the full dataset, client contracts, and ISO audit reports for re-analysis
Comparative observational study^26^	42	1. HCQ 2. SOC	Sig.Dif. in virological cure rates between HCQ (70%) and SOC (12.5%) groups (*P*=0.001)	Selection bias: SOC group comprised Pts who refused HCQ Tx Failure to follow-up Pts for adequate post-treatment periods Age difference between HCQ and SOC groups: mean age 51.2 years vs 37.3 years, respectively (*P*=0.06) Unclear exclusion criteria for HCQ group 5 SOC Pts with no virological test on day 6 were included in the final analysis as COVID-19-positive cases
Re-analysis of study,^26^ including the missing datasets^27^	42	1. HCQ 2. SOC	No significant differences in virological cure rates on treatment days 3, 4, 5, or 6*	None. Study refers to methodological problems of the abovementioned study
Uncontrolled observational study^28^ aimed at replicating the above comparative observational study^26^	11	1. HCQ	20% of patients were virologically cured at day 5–6 post-inclusion*	

* Only primary outcomes are shown, unless the study also provided secondary outcomes.

↑, increased/higher; CQ, chloroquine; ISO, International Organization for Standardization; HCQ, hydroxychloroquine; Pt(s), patient(s); RT-qPCR, reverse transcription polymerase chain reaction; Sig.Dif., significant difference(s); SOC, standard of care; Tx, treatment.

#### Observational studies

Two studies were uncontrolled, non-comparative, observational studies from France that included patients with laboratory-confirmed COVID-19 who were treated with HCQ and azithromycin (AZ), without a control group.^8^,^9^ In the trial of Gautret et al. clinical improvement was noted in 97.5% of patients (*n*=80); all patients were quickly discharged, and a rapid decline in nasopharyngeal viral load was reported.^8^ The second study, by Million et al., noted prolonged viral carriage in 4.4% of patients (*n*= 1,061) and at least one adverse outcome (either death or transfer to an intensive care unit, or hospitalization for at least 10 days) in 4.3%.^9^

In addition to the lack of a control group, there were other major methodological flaws in these two studies. Patients were relatively young, with median ages of 52.5 and 43 years in the first^8^ and second^9^ studies, respectively. Such young patients have a very low risk for COVID-19 complications, even without any treatment, and post-treatment follow-up time was inadequate (in some patients, only 6 days).

Nine additional observational studies compared HCQ, with or without AZ, to control groups that did not receive HCQ.^10^–^18^ Four of these studies showed no significant mortality rate differences for patients receiving HCQ compared to controls.^10^–^12^,^17^ In one study, the risk for all-cause mortality was significantly higher among HCQ-treated patients com-pared to patients not treated with HCQ (adjusted hazard ratio 1.83; 95% CI 1.16–2.89; *P*=0.009); however, mortality was similar when patients treated with HCQ+AZ were compared to control patients (adjusted hazard ratio 1.13; 95% CI 0.8–2.15; *P*=0.28).^13^ In three studies, two of which were relatively large, a significant reduction in mortality rates was observed in the HCQ group compared to the control group.^14^,^15^,^18^ Several inherent methodological flaws are worth mentioning in these trials (Table 1). In one study, for example, the mean age of patients in the treatment group was 5 years higher than in the control group, and there was a major difference between the number of patients receiving steroid treatment (a treatment that has been shown to be beneficial for some patients) in the HCQ group as compared with the non-HCQ groups (74.3% in HCQ+AZ, 78.9% in HCQ alone, 38.8% in AZ alone, and 35.7% in the control group).^15^

Two observational studies compared HCQ to other drugs.^19^,^20^ Kim et al. compared HCQ to lopinavir-ritonavir as a COVID-19 treatment; these authors observed a faster conversion of viral RNA in polymerase chain reaction (PCR)-based assays among patients treated with lopinavir-ritonavir.^19^ Vahedi et al. assessed HCQ as part of a broader treatment regimen containing multiple drugs; no beneficial change in oxygen saturation, body temperature, and C-reactive protein levels were observed.^20^

#### Observational studies with severe methodological issues

Gautret and colleagues conducted an open-label non-randomized trial in France; they found significantly higher numbers of virologically cured patients at 6 days in the group of patients treated with HCQ compared to the control group (70% versus 12.5%, respectively, *P*=0.001).^26^ However, this study had several severe methodological flaws. Firstly, there was a major difference in group size and mean patient ages (26 patients, with mean age of 37.3 years, in the treatment group versus 16 patients, with mean age of 51.2 years, in the control group). Secondly, 6 patients were strangely excluded from the HCQ treatment groups, for example, due to a death that was erroneously defined as “lost to follow-up,” (*n*=1), transfer to an intensive care unit (*n*=3), adverse effects of treatment (*n*=1), and early recovery (*n*=1). Finally, the control group (*n*=16) had no follow-up data for 5 patients on day 6, nor on day 5 for 2 of the same patients, resulting in an exaggeration of estimated COVID-19 positivity.

An independent appraisal study describing a re-analysis of the original Gautret dataset^26^ was recently published by Intson et al.^27^ By excluding all missing datasets, the authors found no differences in viral clearance rates between the treatment and control groups on days 3, 4, 5, or 6. Reproducibility is also a major issue in the Gautret study, as another open-label trial by Molina et al.^28^ used the same dosing regimen and reported that virological cure rates at day 5–6 were only 20%, as opposed to the 70% reported by Gautret et al.

Another unusual occurrence was the retraction of a large multinational registry analysis published in *The Lancet*.^29^ This study contained the data of tens of thousands of patients and demonstrated a significant increase of in-hospital mortality rates among the HCQ-treated group compared to patients not treated with HCQ. The study was retracted at the request of three of the co-authors, following the refusal of Surgisphere Corporation, which initially provided the patient dataset, to transfer the full dataset, client contracts, and International Organization for Standardization (ISO) audit reports for re-analysis.^30^

#### Randomized controlled trials

Two small open-label RCTs comparing HCQ treatment to control patients who did not receive HCQ treatment were conducted in China. Both showed no significant differences in virological cure between the control and treatment groups.^23^,^24^ Chen et al. found no benefits of HCQ treatment in terms of the median time for body temperature normalization, radiological progression on CT images, and clinical improvement^23^; Tang et al. found no significant difference in the virological cure rate on day 28 with HCQ treatment.^24^

A large RCT conducted in Brazil showed no benefit for HCQ-treated patients and reported a higher incidence of QTc prolongation and elevation of liver enzymes among treated patients.^25^

Preliminary results of the large British RECOVERY RCT were recently released to the public before peer review. This study showed no significant differences in the lengths of hospital stay and the death rates at 28 days between the HCQ-treated group and controls.^22^

### Studies Evaluating COVID-19 Prevention with HCQ

Boulware et al. recently published a randomized, double-blind, placebo-controlled trial that assessed hydroxychloroquine use as a post-exposure prophylaxis agent within 4 days of COVID-19 exposure.^31^ The researchers enrolled adults who had household or occupational exposure to someone with confirmed COVID-19, using a strict exposure definition. Participants received either placebo or HCQ, and the primary outcome was the incidence of either laboratory-confirmed COVID-19 or symptoms compatible with COVID-19 within 14 days. Participants were randomized into two groups: HCQ prophylaxis (*n*=414) and control (placebo; *n*=407). The numbers of new COVID-19 cases in the treatment and control groups were 49 and 58, respectively (*P*=0.35). Side effects were more common with HCQ than with placebo (40.1% versus 16.8%), but no serious adverse reactions were reported.

### Studies Evaluating the Safety of HCQ

Most adverse reactions of treatment with HCQ are mild and include gastrointestinal adverse effects (nausea, vomiting, loss of appetite, diarrhea), skin rash, and photosensitivity.^32^ Prolonged treatment has been associated with irreversible dose-dependent toxic retinopathy. Cardiac toxicity is rare and includes heart failure, conduction disturbance, and QT prolongation. QT prolongation occurs with increasing incidence among older patients and those treated with other medications that prolong the QT interval, e.g. AZ.^33^

In this review we focused on HCQ effectiveness in treating COVID-19. However, several observational studies assessed the safety profile of HCQ, mainly focusing on cardiovascular adverse events. The observational study by Rosenberg et al. demonstrated a higher likelihood of cardiac arrest in patients receiving HCQ+AZ (adjusted OR 2.13; 95% CI 1.12–4.05), but not HCQ alone (adjusted OR 1.91; 95% CI 0.96–3.81) or AZ alone (adjusted OR 0.64; 95% CI 0.27–1.56).^10^

A special attention was given to cardiac arrythmias and QT prolongation following HCQ treatment. In a non-comparative observational study, 24 (2.3%) of 1,061 patients treated with HCQ reported mild adverse events and no cardiac arrhythmias.^9^ However, many other studies reported prolonged QT intervals among HCQ-treated patients.^9^,^10^.^24^,^25^,^33^,^34^ Of note, one patient developed polymorphic ventricular tachycardia requiring emergent cardioversion, and seven patients required premature termination of therapy.^35^,^36^ Moreover, in a recently published RCT, rates of QTc prolongation were 14.7%, 14.6%, and 1.7% in patients treated with HCQ+AZ, HCQ alone, or none, respectively.^25^

## DISCUSSION

This review aimed to assess the possible beneficial role of HCQ in prophylaxis and treatment of COVID-19. To that end, we performed a PubMed search and then narrowed our review down to the 17 most relevant studies. Most studies assessing the therapeutic role of HCQ were observational, and many had an inherent risk of bias.^8^–^20^ Overall, these observational trials had inconclusive results. The RCTs that have been published so far point to a lack of benefit, although the results are mixed. A large British study, which is expected to be published soon, showed no benefit of HCQ according to early released data.^22^ Use of HCQ as a prophylactic agent was not beneficial in one well-conducted RCT, indicating that its use for COVID-19 should be abandoned.^31^

The safety profile of HCQ is important to assess considering three observations: the drug has no place in therapy as a prophylactic agent^31^; its therapeutic benefits are questionable^22^,^23^,^25^; and its use has been advocated by key political leaders and on social media platforms.^37^ In the few studies that assessed the adverse events of HCQ, mild gastrointestinal side effects were relatively common. The rate of QT prolongation was quite variable (7%–76%) in the few studies reporting it. Whether this risk is translated into an increased risk for cardiac arrhythmias remains to be seen. Even a relatively small risk of such a major event would translate into a large total number of adverse events, since HCQ, at least in the early days of the COVID-19 pandemic, was administered to tens of thousands of patients.^33^–^36^

Perhaps more important than the assessment of HCQ itself is the critical assessment of events that have led to where we are today. Official guidelines regarding the use of HCQ vary greatly between countries and have changed with time, making the roller-coaster of pros and cons for HCQ use hard to follow. In early March 2020, the Italian Medicines Agency (AIFA) supported the use of chloroquine and HCQ for treatment of COVID-19.^38^ Shortly afterward, a personal statement supporting the use HCQ as a prophylactic and therapeutic agent was issued by the president of the United States of America (USA). The US Food and Drug Administration (FDA) was quick to follow, and issued an emergency use authorization (EUA) to distribute HCQ and chloroquine for the treatment of some COVID-19 in-patients.^39^ By April, however, the FDA cautioned against HCQ and chloroquine use due to ensuing risks of arrhythmias^40^; in May, 2020, an article published in *The Lancet* demonstrated a significant reduction in COVID-19 survival rates following HCQ use.^29^ This article, in turn, was later retracted but only after France, Italy, and Belgium banned the use of HCQ treatment for COVID-19 patients. In June, the preliminary results of the RECOVERY Trial were published demonstrating that HCQ is ineffective in COVID-19 patients compared to control,^22^ and the FDA quickly revoked the EUA for both HCQ and chloroquine.^39^ The US Centers for Disease Control and Prevention is currently recommending against the use of chloroquine or HCQ for the treatment of COVID-19, with the exception of patients included in clinical trials. The final results of a large and well-conducted RCT have still not been made fully public.^41^

Several factors have prevented a quicker and more thorough assessment of HCQ over the past few months. The most astonishing fact, when one considers the urgent need for reliable research during a global pandemic, is the fragmentation of scientific efforts between and within countries. Many low-quality observational trials and very few RCTs were conducted. Even now, 7 months after the first cases were reported from Wuhan, more than 300 clinical trials assessing HCQ for COVID-19 are listed in the clinical trial.gov database. Most of these studies will probably never be completed. Instead of creating a few large national or international RCTs with uniform protocols that could later be meta-analyzed, hundreds of different protocols have been created, wasting time and resources.

Large research networks should be created in advance and activated quickly when the need arises. The yet-unpublished British RECOVERY Trial may be the finest example of such an effort during this pandemic.^22^

Selection bias, i.e. including young and relatively healthy patients who are not expected to die from the infection, was also a major contributor to the rendering of the results of several studies as essentially irrelevant for severely ill COVID-19 patients.^8^,^9^,^14^ The “corona publication rush” led some scientists to submit studies with major methodological flaws. Listing a patient death as a “loss to follow-up” may be an extreme example,^26^ but numerous other methodological issues are seemingly abundant. Observational studies are inherently inadequate when the efficacy of a drug is to be assessed, but even in the few RCTs that were conducted, the randomization and assignment procedures were unclear and may have been biased.^23^,^24^

Some controlled studies compared populations that were different, an inherent limitation of observational studies. For example, a significant difference in the mean age or the proportion of patients receiving steroids (a medication that has been shown to be beneficial to some patients)^37^ may have influenced the results.^15^,^26^ Drug safety assessments, critical to any trial that involves drug administration for a new indication, were absent from some studies. Shortcomings of the peer review procedures of medical journals were evident. On the one hand, slow peer review meant that initial results, often skewed, inconsistent, or just plain wrong, were available on sites such as medRXiv and the social media platforms many weeks before actual publication. On the other hand, efforts to fast-track what was perceived to be important data led to studies with major methodological flaws being accepted for publication,^26^ and other studies were accepted only to be retracted later. It is crucial for studies that assess data, which could have a major immediate impact on patient management, to be peer-reviewed with a strict protocol that ensures both speed and quality.

The scientific community possesses unprecedented powerful research capabilities owing to cutting-edge technologies, global data sharing, and computational analysis capabilities never previously seen. In theory, these advances should have enabled the scientific community to answer what is, ultimately, a very simple question: is hydroxychloroquine helpful for treating COVID-19? The answer for HCQ as prophylaxis for COVID-19 has been adequately answered, albeit with a no-benefit conclusion. Is hydroxychloroquine beneficial for the treatment of millions of COVID-19 patients? Probably not, but sadly, more than 7 months and 600,000 deaths into the pandemic, that question still has not been definitively answered.

## Abbreviations

AZ: azithromycin
COVID-19: coronavirus disease 2019
EUA: emergency use authorization
FDA: US Food and Drug Administration
HCQ: hydroxychloroquine
ICU: intensive care unit
ISO: International Organization for Standardization
RCT: randomized controlled trial
SARS-CoV-2: severe acute respiratory syndrome coronavirus-2
